# Real-Life/Real-Time Elderly Fall Detection with a Triaxial Accelerometer

**DOI:** 10.3390/s18041101

**Published:** 2018-04-05

**Authors:** Angela Sucerquia, José David López, Jesús Francisco Vargas-Bonilla

**Affiliations:** 1 Facultad de Ingeniería, Institución Universitaria ITM, Cra. 65, 98A-75 Medellín, Colombia; 2 SISTEMIC, Facultad de Ingeniería, Universidad de Antiquia UDEA, Calle 70, No. 52-21 Medellín, Colombia; josedavid@udea.edu.co (J.D.L.); jesus.vargas@udea.edu.co (J.F.V.-B.)

**Keywords:** triaxial accelerometer, wearable devices, fall detection, mobile health-care, SisFall, Kalman filter

## Abstract

The consequences of a fall on an elderly person can be reduced if the accident is attended by medical personnel within the first hour. Independent elderly people often stay alone for long periods of time, being in more risk if they suffer a fall. The literature offers several approaches for detecting falls with embedded devices or smartphones using a triaxial accelerometer. Most of these approaches have not been tested with the target population or cannot be feasibly implemented in real-life conditions. In this work, we propose a fall detection methodology based on a non-linear classification feature and a Kalman filter with a periodicity detector to reduce the false positive rate. This methodology requires a sampling rate of only 25 Hz; it does not require large computations or memory and it is robust among devices. We tested our approach with the SisFall dataset achieving 99.4% of accuracy. We then validated it with a new round of simulated activities with young adults and an elderly person. Finally, we give the devices to three elderly persons for full-day validations. They continued with their normal life and the devices behaved as expected.

## 1. Introduction

At least one-third of elderly people suffers a fall per year and the probability of falling increases as age and the number of previous falls increase [1,2,3,4]. The consequences of a fall can be reduced if the person is attended by medical services within an hour of the accident [5,6,7]. This timing is feasible with institutionalized elderly people, but healthy independent elderly people are often alone for long periods of time, increasing their risk of aggravating the injuries in case of an accident. Researchers now focus on developing automatic fall detection systems that generate an alarm in case of an event, but they still present high error rates (see [7,8,9,10] for reviews in the field). In this paper, we tackle this issue with a novel fall detection methodology validated under real-life conditions with the target population. Our approach is based on a non-linear classification feature, with only one triaxial-accelerometer for acquisition, a 25 Hz sampling frequency, and a gait detector for false positives avoidance.

Detecting falls with a triaxial accelerometer is commonly divided in three stages: pre-processing, feature extraction, and classification. The preprocessing can be as simple as a low-pass filter [11], but this stage mainly depends on the features selected for extraction. Many features can be extracted, including acceleration peaks, variance, and angles (see [9] (Table 4) for a complete list). These features transform the acceleration signal in order to better discriminate between falls and activities of daily living (ADLs). Regarding classification, threshold-based detection is still preferred over machine learning alternatives, mainly because the latter are impractical for real-time implementation. Habib et al. [10] show various examples of support vector machine (SVM)-based approaches that consume the battery in a few hours, and Igual et al. [12] concluded that these approaches are highly dependent on the acquisition device used.

A common problem with approaches proposed in the literature is that most of them have been tested with young adults under controlled conditions [9] (Table 5). Moreover, previous works have demonstrated that the accuracy of these approaches is significantly reduced when tested on institutionalized [13] and independent [11] elderly people. The main reason authors have for not testing with the target population is the lack of appropriate public datasets and the difficulty of acquiring real falls with elderly people [9,11,13]. In order to tackle these issues, we recently released the SisFall dataset [11], a fall and movement dataset acquired with a triaxial accelerometer mounted on an embedded device attached to the waist (see [14] for implementation details).

In [11], we demonstrated that most failures in fall detection are focused on a few activities. Most of these activities coincide in periodic waveforms (from walk and jog) and high peak acceleration ADLs (e.g., jump). Previous approaches in the literature involve detecting jog and walk with accelerometers. Cola et al. [15] detected gait deviation as a fall-risk feature. Oner et al. [16] used peaks of the acceleration signal measured with a smartphone to detect steps and subsequently the kind of activity based on the period between steps. Wundersitz et al. [17] achieved this with an embedded device. Other authors have used more elaborated metrics, but all have been peak-based. Clements et al. [18] computed principal components of the fast Fourier transform (FFT), to cite an example. In contrast, we previously developed a more stable gait detector based on wavelet or auto-correlation indistinctly [19]. However, it was too computationally intensive for real-life implementation in an embedded device.

In this work, we present a Kalman-filter-based fall detection algorithm that additionally detects gait as a feature to avoid false positives. The fall detection feature is a novel non-linear metric based on two widely used features: the sum vector magnitude and the standard deviation magnitude. The Kalman filter is a well-known optimal estimator [20] widely used in several research fields. The Kalman filter is Markovian (avoiding large memory storage) and linear (simple computations for lower energy consumption). Here, we use it as an input to the non-linear feature by determining the orientation of the subject: jogging activities may lead to high accelerations, but the absence of inclination implies that the subject is not falling. We additionally use the Kalman filter to smooth gait patterns (as sinusoidal-shape waveforms) in order to feed our gait detector.

The Kalman filter has been previously used to identify movements of interest with accelerometers. Bagalà et al. [21] used it to determine the lie-to-sit-to-stand-to-walk states, which are commonly used to measure the risk of falling in elderly people (with the Berg Balance Scale –BBS– for example [22]). There, the authors used an extended Kalman filter to determine the orientation of the device. Otebolaku et al. [23] proposed a novel user context recognition using a smartphone. In their work, the Kalman filter was used to obtain the orientation of the device based on its multiple sensors (not only the accelerometer). However, the authors did not specify how they achieved this. Novak et al. [24] used a multiple-sensor system to determine gait initiation and termination. In their work, the Kalman filter was used again to obtain the orientation of the device.

The aforementioned works coincide in their objective with the Kalman filter (identifying locomotion activities), but they differ in the way it was implemented, and it was never used to detect falls. Other authors have used the Kalman filter as part of their fall detection algorithms [25,26,27,28,29]. All of them used the triaxial accelerometer together with other sensors (a gyroscope in all cases and a magnetometer in one case). In these works, the Kalman filter was used for de-noising and data fusion, and to obtain the device angle. The main difference among these works is the classification strategy: a threshold, an SVM, neural networks, or a Bayesian classifier.

Our approach differs from these previous works in several key features: (i) We present a novel non-linear classification feature that allows one to obtain high accuracy values with a simple thresholding approach; (ii) We only use one triaxial accelerometer so as to extend the battery life of the device. We did not use a gyroscope because it typically consumes more than 10 times more current than the accelerometer (30–140 μA for the ADXL345 that we selected, compared to a range from hundreds of μA to a few mA required by a gyroscope); (iii) We include a gait detector to discard false positives caused by high acceleration periodic activities; (iv) We validated our approach in real time and under uncontrolled conditions with the target population.

Using a single accelerometer with a threshold-based fall detector has traditionally been presented as a low-accuracy alternative compared to multi-sensor SVM approaches. In this paper, we demonstrate that our novel non-linear metric, together with a Kalman filter smoother and a simple zero-crossing gait detector, achieve an up to 99.4% accuracy on the largest dataset freely available in the literature. This paper is divided as follows: In Section 2 and Section 3, we present the datasets used and explain the proposed approach. In Section 4, we present the overall results with controlled activities and falls (including real-time implementation on an embedded device), we analyze individual activity, and we show an on-line validation, where three elderly voluntaries each carried an embedded device for several days. Finally, we discuss our findings in Section 5.

## 2. Materials

We recently published a dataset with falls and ADLs acquired with an accelerometer (SisFall: Sistemic research group fall and movement dataset [11]). Here we use this dataset to train and test the proposed approach. It was generated with 38 participants comprised of both elderly people and young adults. Twenty-three young adults performed five repetitions of 19 ADLs and 15 fall types, while 14 participants over 62 years old performed 15 ADLs. One additional participant, a 60-year-old participant, performed both ADLs and falls. The dataset was acquired with a self-developed embedded device attached to the waist [14]. The embedded device was based on a Kinets MKL25Z128VLK4 microcontroller with an ADXL345 accelerometer. The accelerometer was configured for ±16 G, 13 bits of ADC, and a sampling rate of 200 Hz.

**Figure 1:**
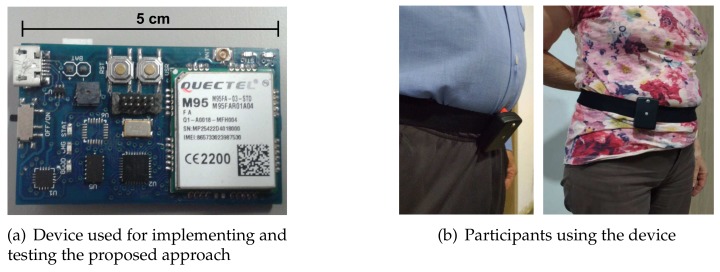
Validation device. (**a**) With similar characteristics of the device used in [11], this one included a GPRS module able to send text messages in case of alarm; (**b**) Note how the elderly participants did not wear the device exactly on the expected position/orientation (The photos show positions observed at the end of uncontrolled recordings).

A second device was developed for validating our methodology (Figure 1). This device consisted of the same microcontroller and sensor used for SisFall, but it included a GPRS transmitter (to send short text messages—SMS) that was activated if a fall was detected. As we did with the first device, it was fixed with a homemade belt (see the supplementary videos of [11]) to guarantee that it did not move relative to the subject. The device did not need to be in a completely vertical position, nor did it require additional calibration once the subject was wearing it.

Two additional validation tests were performed with this device:

- Individual activities: Six young adults (subjects SA03, SA04, SA05, SA06, SA09, and SA21) and one elderly person (subject SE06) performed again three trials of all activities in SisFall (except for D17, getting in and out of a car, due to logistic issues).
- On-line uncontrolled tests: We gave the device to three elderly participants that were not part of the SisFall dataset. They were independent and healthy. Table 1 shows their gender, age, height, and weight. The subjects used the device permanently for several days, except while sleeping and showering (as the device is not waterproof yet). We used three devices to guarantee the integrity of the system.

All activities performed by the participants were approved by the Bio-ethics Committee of the Medicine Faculty, Universidad de Antioquia UDEA (Medellín, Colombia). Additionally, all participants were evaluated by a sports specialized physician.

## 3. Methods

**Figure 2:**
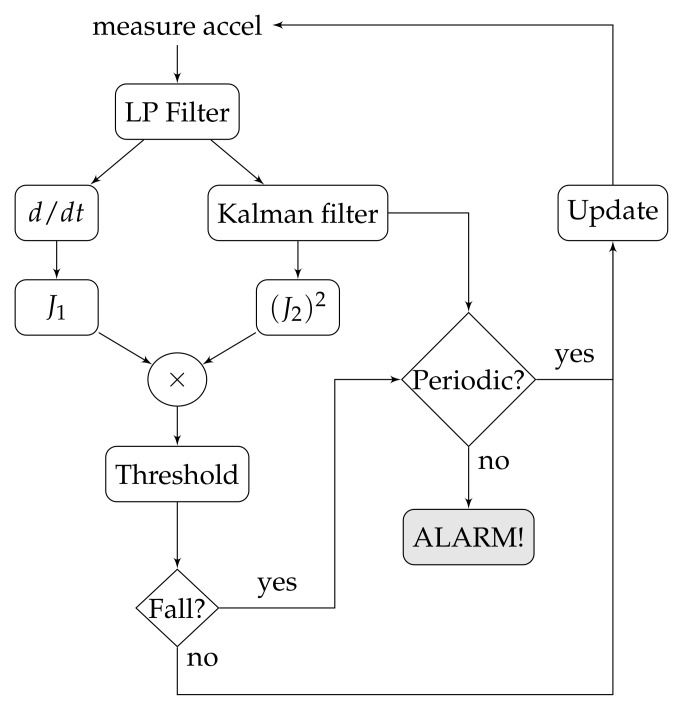
The proposed methodology is based on a non-linear feature that allows for fall detection with a simple threshold-based detector. False positives are then discarded if a periodic activity is detected after the fall.

Figure 2 shows a schematic of the proposed approach. It includes bias variations of the signal together with acceleration peaks. This increases the robustness of the feature extraction and allows for simpler classifiers. The proposed methodology consists of four stages: preprocessing, feature extraction, classification, and periodic activity detection. For each time sample *k*, the raw acceleration data $\vec{a}\left[k\right]$ is initially low-pass-filtered. It then splits into bias removal and Kalman filtering, which feeds both features $J_{1}$ and $J_{2}$, respectively (see Equations (8) and (9) below). A threshold-based classification is performed over a non-linear indirect feature. If the resultant value crosses the threshold, the periodicity of the signal (extracted from the Kalman filter and a zero-crossing algorithm) is analyzed in order to determine if it is a false fall alert or if indeed the alarm should be turned on. This methodology is explained in the following section.

### 3.1. Pre-Processing Stage

The 4-th order IIR low-pass Butterworth filter with a cut-off frequency of 5 Hz proposed in [11] was used in this work. This filter was selected for the following reasons: (i) It can be implemented in simple embedded devices; (ii) It does not require large computations in software; (iii) An increase in the order or in the cut-off frequency does not improve the accuracy. Consequently, it does not require higher sampling frequencies. Filtered data are then bias removed with a simple differentiation of consecutive samples, as is needed to compute the static feature ($J_{1}$). The SisFall dataset was initially acquired at 200 Hz; however, the proposed methodology only requires 25 Hz to feed the filter. All results presented here correspond to the proper downsampled signals.

The second feature ($J_{2}$) is computed over the bias level, which is obtained with a Kalman filter. A Kalman filter [20] is an optimal quadratic estimator able to recover hidden states of a state-space model. It was used here with two purposes: to recover the bias-level variation and to find the periodicity of the signal. To achieve this, the Kalman filter acts as a set of adaptive FIR filters with the objective of estimating parameters/states of a dynamical system. As any Luemberger-type estimator [30], the estimation is improved by working in a closed loop, which allows for updating the input and using the historic values collected on the Markovian process. Additionally, being a multivariable estimator, the Kalman filter includes both the variance and covariance of the signal (something unfeasible with FIR filters). Finally, as an adaptive filter, it has more immunity to model uncertainty, noise, and perturbations than FIR filters (it is optimal on the quadratic mean, and it is robust).

**Figure 3:**
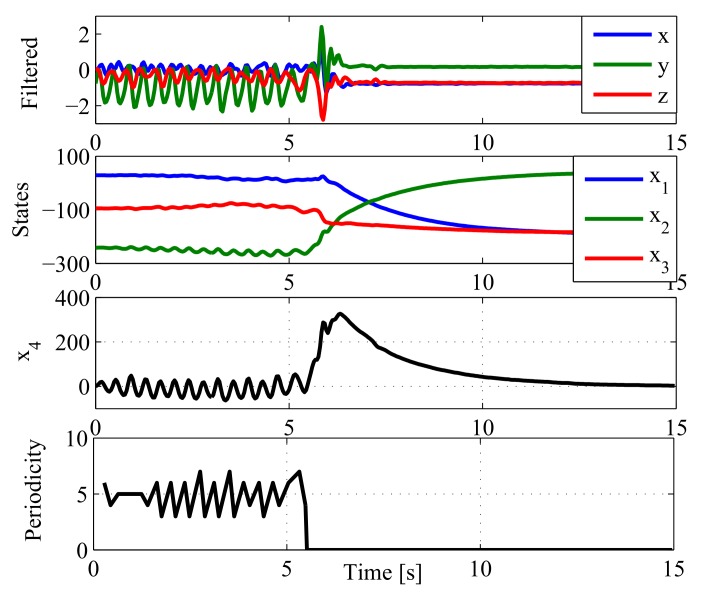
Kalman filtering. (**top panel**) Reference filtered acceleration data (Activity F05 of SisFall: jog, trip, and fall) in gravities [G]. (**second panel**) First three states of the Kalman filter. The filter estimates the bias-level variations of the signal. (**third panel**) The fourth state of the Kalman filter recovers a quasi-sinusoidal signal during the first 6 s. Its objective is to dynamically remove bias to allow posterior zero crossing detection. (**bottom panel**) Periodicity detector. In the first 6 s, the subject is jogging with a period of 10 time samples (half zero crossings); when the subject suffers a fall, it stops detecting periodicity too.

Let us define the filtered acceleration data as $\vec{a}\left[k\right]={\left[a_{x},a_{y},a_{z}\right]}^{T}\in {ℜ}^{3\times 1}$ for time instant *k*, where $a_{x}$, $a_{y}$, and $a_{z}$ are single samples of raw acceleration (in practice, it comes in bits, as acquired by the ADC of the device). These data feed the following autonomous state-space model:

(1) $$\begin{array}{cc} \vec{x}\left[k\right] & =A\vec{x}\left[k-1\right]+\eta \\ \vec{y}\left[k\right] & =C\vec{x}\left[k\right]+\epsilon \end{array}$$

where the first three states of $\vec{x}\in {ℜ}^{4\times 1}$ are used for classification, and the fourth state $x_{4}$ removes peaks from periodic signals (see Figure 3 for an example with activity F05: jog, trip, and fall). As this Kalman filter is exclusively used for smoothing (and not for feature extraction or classification), the state transition $A\in {ℜ}^{4\times 4}$ and output $C\in {ℜ}^{4\times 4}$ matrices are identity matrices. Finally, the output is defined as $\vec{y}={\left[a_{x},a_{y},a_{z},a_{y}-b_{a_{y}}\right]}^{T}\in {ℜ}^{4\times 1}$, where the first three terms are the low-pass-filtered acceleration data in the three axis, and the fourth output is the acceleration on the vertical axis minus its current bias $b_{a_{y}}$. The vertical bias $b_{a_{y}}\left[k\right]$ is dynamically updated with a sliding window of 1 s over the mean of the output of the Kalman filter. $x_{4}$ provides a zero-bias sinusoidal-shape waveform when the acceleration comes from periodic activities (walk, jog, going-up stairs, etc.). The period of this signal can be detected by counting zero-crossings (changes of sing) and dividing it by two over a given time window.

This state-space model is affected by Gaussian measurement noise ϵ=N(0,R), and Gaussian state uncertainty η=N(0,Q). The objective of the Kalman filter is to minimize the variance of the states $P\in {ℜ}^{4\times 4}$, considering them as random variables with a Gaussian distribution: $\vec{x}=N\left(\bar{x},P\right)$.

The Kalman filter consists of five equations divided in two stages. The prediction stage of the Kalman filter predicts the current value of the states and their variance solely based on their previous values:

(2) $$\begin{array}{cc} \vec{x}{\left[k\right]}^{-} & =A\vec{x}\left[k-1\right] \end{array}$$

(3) $$\begin{array}{cc} P{\left[k\right]}^{-} & =AP\left[k-1\right]A^{T}+Q. \end{array}$$

Both $\vec{x}{\left[k\right]}^{-}$ and $P{\left[k\right]}^{-}$ are intermediate values that must be corrected based on current data values:

(4) $$\begin{array}{cc} G[k] & =CP\left[k\right]{\left(CP{\left[k\right]}^{-}C^{T}+R\right)}^{-1} \end{array}$$

(5) $$\begin{array}{cc} \vec{x}\left[k\right] & =\vec{x}{\left[k\right]}^{-}+G\left[k\right]\left(\vec{y}\left[k\right]-C\vec{x}{\left[k\right]}^{-}\right) \end{array}$$

(6) $$\begin{array}{cc} P[k] & =\left(I_{4}-G{\left[k\right]}^{T}C\right)P{\left[k\right]}^{-} \end{array}$$

where $I_{4}\in {ℜ}^{4\times 4}$ is a (4×4) identity matrix.

This strategy only requires sintonizing two parameters when the Kalman filter is set up: the variance matrices *Q* and *R*. There are no rules to determine their values, but they are not difficult to define of this particular problem. Both are usually diagonal (no interaction among states), and large values of *Q* and *R* tend to the original data: $\vec{x}\approx \vec{y}$, and they are also complementary, i.e., the states are flattened when any of them are reduced. As shown in Figure 3 (second and third panels), the first three states are flat (inclination of the subject), and the fourth one seeks periodic (sinusoidal shape) waveforms.

The states can be initialized with zero values, and P[0]=Q, i.e., selecting uninformative priors. However, for faster convergence $x_{2}\left[0\right]$ and $b_{a_{y}}\left[0\right]$ can be initialized with −1 G (approximately 258 bytes for the device configuration used here), which is the initial condition of the accelerometer in our device. *Q* and *R* can be computed with a simple heuristic process: Using a walk and fall file for testing, (i) initialize *Q* and *R* with identity matrices; then, (ii) for the first three states (first three diagonal values of *Q* and *R*), start an iterative process by reducing the standard deviation (square of each diagonal value) of *Q* by 10 and then doing the same with *R*, until the states start to appear flat. A good fit can be made by reducing the scale to 5 and then to 2, although these matrices show poor sensitivity, i.e., the algorithm can work with approximate values. Finally, (iii) for the fourth state, reduce *Q* and *R* (as in the previous step) until $x_{4}$ shows a sinusoidal shape in periodic activities (walk and jog). The objective is to clean peaks near zero. Preliminary tests (not shown here) demonstrated that the accuracy is not affected by small variations on these parameters. The final values used in this work were

(7) $$\begin{array}{cc} Q=0.001^{2}\times I_{4} & \;R=\left[\begin{array}{cccc} 0.05^{2} & 0 & 0 & 0\\ 0 & 0.05^{2} & 0 & 0\\ 0 & 0 & 0.05^{2} & 0\\ 0 & 0 & 0 & 0.01^{2} \end{array}\right]. \end{array}$$

These values are approximated to those obtained by He et al. [27] for setting-up their Kalman filter. They applied an auto-regressive model to determine *A* (their final value was almost an identity matrix), *Q*, and *R*. In practice, all computations in both the computer (Matlab, Mathworks) and the embedded device are performed in bits and not in gravities to reduce the computational burden.

### 3.2. Feature Extraction

**Figure 4:**
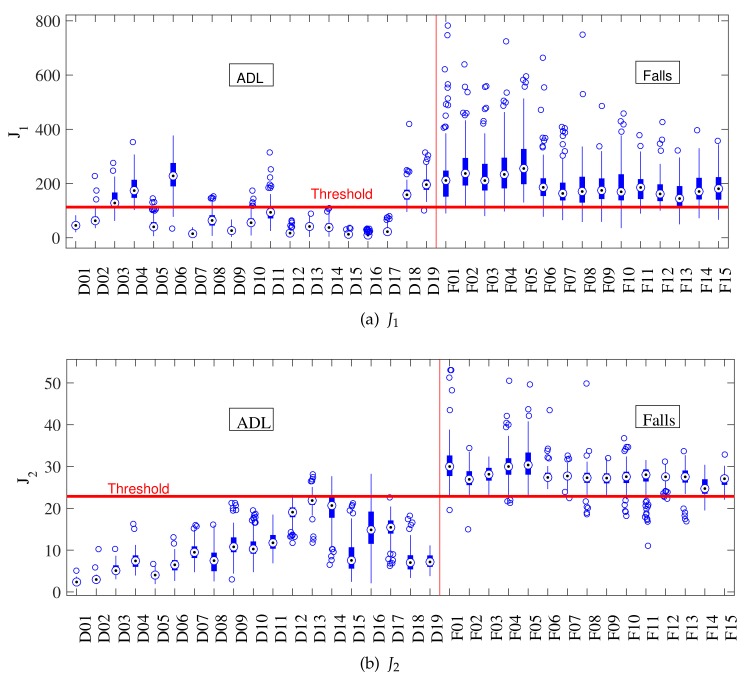
Individual activity analysis of sum vector magnitude $J_{1}$ and standard deviation magnitude $J_{2}$. The horizontal red line corresponds to the optimal threshold value, and the vertical one separates ADLs and falls. (**a**) $J_{1}$ has large errors on periodic activities (D1–D6, D18–D19); while (**b**) $J_{2}$ fails in those that change the body angle (D13, D14).

The main problem of threshold-based approaches is that, among the large number of features proposed in the literature [9] (Table 4), none of them is discriminant over a large set of activities. A preliminary analysis performed with the SisFall dataset [11] indicated that the risk of false positives on most features is higher in two sets of activities: high amplitude periodic activities and transitions between positions (abrupt orientation changes). Periodic activities comprise walking, jogging, and walking up- or downstairs, while transitions refer to all changes among different positions, e.g., standing up from a chair and changing side on a bed. Albeit many fall detection features are weak on similar activities, forming box-plots (such as those of Figure 4) would facilitate determining which of them do not intercept (fail on same activities). This is the basis of our approach.

From the large set of available fall detection features, we have selected for this work two traditional ones that do not fail on the same activities: the sum vector magnitude and the standard deviation magnitude. The sum vector magnitude $J_{1}$ is computed as the root-mean-square (RMS) of the static acceleration with previous bias removal:

(8) $$J_{1}\left[k\right]=\mathrm{RMS}\left(\vec{a}\left[k\right]-\vec{a}\left[k-1\right]\right)$$

where the bias is rejected with differentiation. The standard deviation magnitude $J_{2}$ is computed at each time step *k* over a 1 s sliding window of the first three states of the Kalman filter: $\tilde{x}\left[k\right]=\left[\vec{x}\left[k-N\right],\ldots ,\vec{x}\left[k\right]\right]\in {ℜ}^{3\times N}$, where N=25 is the size of the window (for a frequency sample of 25 Hz):

(9) $$J_{2}\left[k\right]=\mathrm{RMS}\left(\mathrm{std}\left(\tilde{x}\left[k\right]\right)\right)$$

where std(·) is the standard deviation operator. The size of the window is selected as the one that includes the three stages of the fall: pre-fall, the impact, and the period that immediately follows [31]. Testing with windows between 0.25 and 2 s did not improve the accuracy, as expected [11]. The same sliding window can be used to determine the current bias on the *y* axis: $b_{a_{y}}\left[k\right]=\mathrm{mean}\left({\tilde{x}}_{y}\left[k\right]\right)$.

Figure 4 shows activity-by-activity analysis boxplots of $J_{1}$ and $J_{2}$. The horizontal red line is the threshold for the best accuracy value and the vertical red line divides ADLs and falls. The indexes of the activities can be found on [11] (Tables 1 and 2). By comparing $J_{1}$ (Figure 4a) and $J_{2}$ (Figure 4b), we observe that $J_{1}$ largely fails in periodic ADLs (D03, D04, D06, D18, and D19 from [11] (Table 2)), while $J_{2}$ does not. $J_{2}$ advances closer to the threshold in activities where $J_{1}$ does not (D16 for example).

The principle that we use to form our novel non-linear feature is simple: by multiplying those features that do not fail on the same activities, the resultant feature is robust against all of them. Large values on both candidate features will be large on the final metric (fall), and mixed values will stay below the threshold (ADLs). A square on the most accurate feature will prioritize it. Our approach can be implemented by following these steps:

1. Generate an ADL/fall box-plot for each candidate feature, as shown in Figure 4a,b.
2. For each feature, draw a threshold computed following the maximum accuracy of the feature.
3. From visual inspection, select those features that fail in separated activities.
4. Multiply the selected features. Test with and without the square of the most accurate feature and select the option with higher performance.

**Figure 5:**
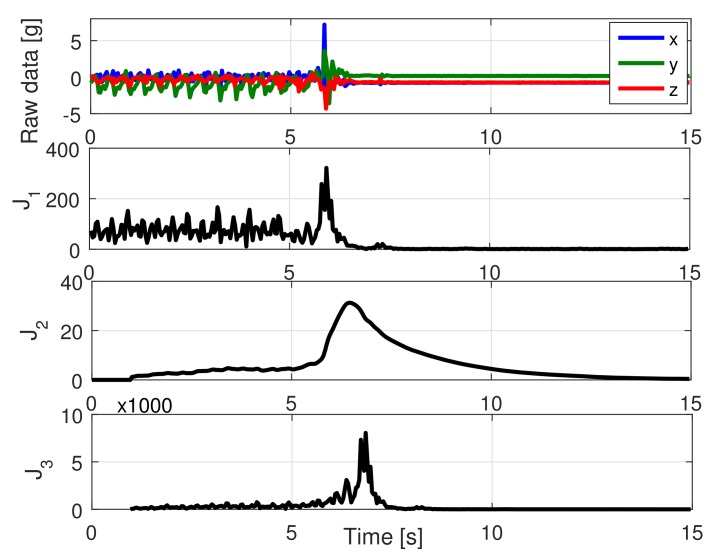
Feature extraction. (**top panel**) Reference raw data (the subject is running, trips, and falls). (**second panel**) Feature $J_{1}$ detects the fall as a large difference between its peak and jogging peaks. (**third panel**) Feature $J_{2}$ has a similar shape but with a larger percentual difference. Both $J_{1}$ and $J_{2}$ are computed in bits for reducing computations on the embedded device. (**bottom panel**) $J_{3}$ is formed by $J_{1}$ and $J_{2}$, increasing their coincidences and diminishing their differences.

Following this approach, our feature extraction consists of a non-linear feature composed of $J_{1}$ and $J_{2}$ as follows:

(10) $$J_{3}\left[k\right]=\max\left({\tilde{J}}_{1}\left[k\right]\right)\cdot \max{\left({\tilde{J}}_{2}\left[k\right]\right)}^{2}$$

where ${\tilde{J}}_{i}\left[k\right]\in {ℜ}^{N\times 1}$ is a sliding window with the last *N* values of the corresponding feature. This window is necessary as the Kalman filter takes some time to achieve the maximum, i.e., both metrics do not always present a maximum at the same time. The objective of this product of features is to amplify the values of those activities where both features agree and to minimize those where both features disagree (see Figure 5, bottom panel). The square of $J_{2}$ prioritizes it over $J_{1}$, as it is more accurate [11]. Figure 5 shows both features with the jog–trip–fall example of Figure 3. The maximum values during jogging are half of those of the fall of $J_{1}$, but clearly become distant in $J_{2}$.

### 3.3. Periodicity Detector

A close analysis of Figure 4a indicates a higher number of false positives in $J_{1}$ for periodic activities (D1–D6 and D18–D19: walking, jogging, etc.) compared with transitional ones (D7 to D17: sitting on a chair, lying on a bed, etc.). This is due to the high acceleration inherent to periodic activities. Additionally, periodic activities are commonly considered as single events in most databases (such as transitions). However, from all activities with a risk of falling, walking is the one that is performed more frequently and for a larger time (see Section 4.4), i.e., the activity with a higher risk of falling. Additionally, trips without falling may increase the probability of having a false positive. Therefore, we developed a periodicity detector aiming to increase the robustness of our fall detector to this specific set of activities. Its objective is to determine whether a fall detected on a periodic activity is indeed a false alarm. If a person falls, it is not expected to continue detecting a periodic signal; therefore, if it does, a false alarm can be concluded.

We focused on a robust and non-computationally expensive alternative for detecting the period of the *y*-axis acceleration signal (vertical axis). We first filter the signal and remove its bias with the Kalman filter, obtaining a quasi-sinusoidal waveform $x_{4}$ when the subject is performing periodic activities. The bias removal is dynamic, as it is critical for the zero-crossing detection used to determine the period. We determine a sign change by subtracting two consecutive samples of $x_{4}$. Once a zero-crossing is detected, the number of samples prior to a new zero-crossing are recorded. The period is simply twice the number of samples recorded. The detailed implementation of the periodicity detector is presented as follows:

1. At time step *k*, the current vertical bias level $b_{a_{y}}\left[k\right]$ is determined by averaging a sliding window of 1 s over $a_{y}$.
2. State $x_{4}\left[k\right]$ of the Kalman filter is then tuned to eliminate local maxima and minima when the shape of the acceleration signal is close to a sinusoid (characteristic of periodic activities). Simultaneously, the current bias level $b_{a_{y}}$ is removed from $x_{4}\left[k\right]$. Figure 3 (bottom panel) shows how state $x_{4}$ tends to a zero-bias sinusoidal shape when the person walks or jogs.
3. A simple zero-crossing periodicity detector is implemented. It consists in determining the number of data samples between each change of sign on $x_{4}$, and multiplying this value by two.
4. The periodicity detector analyzes three seconds after a possible fall event. If during this 3 s window, the periodicity is kept stable, we may expect that it was not a fall. The size of the window is selected as the minimum needed to determine if the person is slowly walking. Note from Figure 3 (bottom panel) how the periodicity is lost when the person trips and falls.

### 3.4. Classification

The classification consists of a single threshold over $J_{3}\left[k\right]$ computed at each time step *k*. The value of the threshold is defined after a training process. The robustness of the threshold was analyzed with a cross-validation set-up. This analysis was performed guaranteeing the same proportion of falls and ADLs in all groups (4510 files randomly divided in 10 groups). A 10-fold cross-validation was performed, and each fold had 4059 files for training and 451 for validation. Each group was used in one round as validation data.

Considering that most falls used for training come from young adults, we have taken in consideration two facts observed in [11]: (i) The elderly adults show on average lower accelerations in both ADLs and falls (this behavior was originally studied in [32]); (ii) The elderly adult that simulated falls always tried harder than the young adults to avoid injury when falling, which is what one would expect from someone having an accident. These two facts lead to a single recommendation: if there is a range for selecting the threshold, the lower acceleration value should be selected to avoid false negatives.

Accuracy (ACC), sensitivity (SEN), and specificity (SPE) were used as performance metrics. SEN and SPE were calculated as specified in [33]:

(11) $$\mathrm{SEN}=\frac{TP}{TP+FN}\;\mathrm{SPE}=\frac{TN}{TN+FP}$$

where TP is the number of falls correctly classified, FN accounts for falls that the algorithm did not detect, TN is the number of ADLs correctly classified, and FP indicates false falls. The accuracy was calculated using Equation (12):

(12) $$\mathrm{ACC}=\frac{\mathrm{SEN}+\mathrm{SPE}}{2}.$$

This balanced computation of the accuracy is selected due to the large difference between the number of ADL and fall files.

### 3.5. Power Consumption

This methodology presents several advantages in terms of power efficiency:

- Sampling frequency: Working directly with the inclination of the subject and not with the peak of the fall allows for reduction in sampling frequency from the usual 50–100 Hz to just 25 Hz. Considering that the fall detection algorithm must be computed every time a new sample arrives, the computation time required in other methodologies is halved. In terms of power consumption, this means that the device will be in an idle state for longer time periods, with a consequent reduction in battery consumption.
- Number of sensors: There is a large difference in power consumption between an accelerometer and a gyroscope (with wide variations differing between references, but with the same trend). The gyroscope consumes on average between 6 and 10 times more current than an accelerometer (the ADXL345 selected for this work consumes 30–140 μA according to its data sheet). Table 2 shows the current consumption (under normal operating modes) of commercial embedded gyroscope and accelerometer sensors (obtained from their respective data sheet).
  Assuming that the fall detection algorithm consumes a similar amount of current with or without a gyroscope, we can estimate the reduction in the battery charge on the same scale; i.e., a device without a gyroscope (like ours) could stay active 5–10 times longer than a device with one.
- Threshold-based classifier: In recent years, authors have focused on machine-learning-based classifiers. The reason is clear: though a large amount of features can be extracted (see [9] (Table 4)), none of them has proven to be discriminant enough. We have powered our discrimination feature by non-linearly combining well-known metrics. Our approach, although simple, allowed us to go back to a simple threshold classifier, which significantly reduces the power consumption compared, for example, with SVM alternatives [10].

## 4. Results

### 4.1. Fall Detection

We initially tested the performance of the proposed algorithm without detecting periodic activities. Table 3 shows the validation results with the SisFall dataset over a 10-fold cross-validation (451 non-repeated files each, which is the same proportion of ADLs and falls). All subjects and activities available in the dataset were included in the cross-validation. The low detection accuracy obtained with $J_{1}$ (around 86%) raises questions as to its utility. However, $J_{3}$ is significantly higher than $J_{2}$ (99.3% vs. 96.5%). Although $J_{1}$ is not a good metric, combined with $J_{2}$, it improves the individual accuracy values.

**Figure 6:**
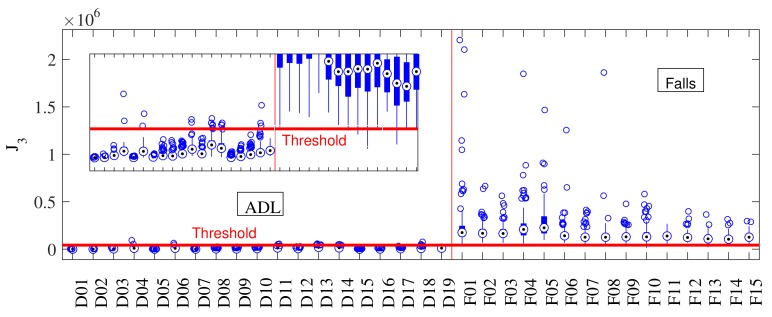
Performance of $J_{3}$ when tested with SisFall. The horizontal red line corresponds to the optimal threshold value, and the vertical one separates ADLs and falls. The combination of $J_{1}$ and $J_{2}$ provided $J_{3}$ with a better discriminant capability (the small box at the left shows a vertical zoom).

Figure 4 and Figure 6 show an activity-by-activity analysis. The horizontal red line is the threshold for the best accuracy value and the vertical red line divides ADLs and falls. After the analysis provided for $J_{1}$ and $J_{2}$ in Figure 4, it was expected that the non-linear combination proposed to form $J_{3}$ would increase their strengths and minimize their weaknesses (sets of activities prompt to fail). The small box in Figure 6 shows how all activities are more separated from the threshold and, more important, fewer fall files crossed the threshold (false negatives) than with $J_{1}$ and $J_{2}$. This initial result significantly improves those obtained with previous approaches tested in [11] (none of them achieved more than 96%).

We performed an additional test without including the Kalman filter in order to determine its effect on the algorithm. As expected, the accuracy of all metrics was significantly reduced: 82.65% for $J_{1}$, 91.03% for $J_{2}$, and 83.73% for $J_{3}$. However, this is not a fair comparison. The Kalman filter could be replaced by a set of band-pass filters, and similar results should be obtained. However, this strategy could severely affect the computational effort of the embedded device and its battery consumption.

### 4.2. Fall Detection with Periodicity Detector

We then performed the same analysis with the periodicity detector. The main purpose of this detector is to take $J_{1}$ to zero if a periodic activity is observed after a possible fall (false positive), and the same result is obtained if $J_{2}$ is selected. Table 4 shows the validation results after a 10-fold cross-validation. Compared to the previous analysis, $J_{1}$ shows an 8% improvement (94.32%). Although one would expect a similar improvement in $J_{3}$, this is not the case (although it is higher, with a 99.4% accuracy) provided that on the SisFall dataset, walk and jog only have one file per subject. Nevertheless, the periodicity detector was active in 606 files (13.5% of the dataset).

**Figure 7:**
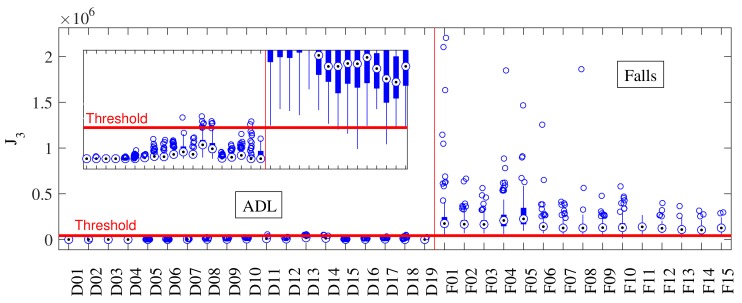
Individual activity analysis of the proposed algorithm including the periodicity detector. The horizontal red line corresponds to the optimal threshold value, and the vertical red line separates ADLs and falls. $J_{3}$ was turned to zero in all periodic ADLs, which allowed it to increase the distance between most ADLs and falls.

Every dataset has a limited number of repetitions per activity. SisFall for example contains only one 1 min repetition of walk per subject. However, it is expected that a walk will last more than a minute, i.e., the possibility of failure is higher with activities that the subject performs regularly (such as walking). Additionally, Figure 7 shows how the possibility of errors in other activities is lower due to their larger distance from the threshold.

Figure 7 shows the same individual activity analysis of Figure 6 but with the periodicity detector in $J_{1}$. Figure 7 shows how Activities D01–D04 were turned to zero, as the detector confirmed that the subject was walking or jogging. In this case, $J_{3}$ shows more distance from the threshold than on the previous test (the threshold is updated accordingly). This indicates that, even the cross-validation did not show a significant improvement on accuracy, the inclusion of the periodicity detector increased the robustness of the algorithm. Importantly, no fall was turned to zero in Figure 7, indicating that the periodicity detector was turned off in all periodic activities that finished in a fall.

We performed a Kappa test between falls and ADLs to statistically verify the robustness of our approach. The procedure consisted in computing the confusion matrix for each fold and then using the mean to compute the observed and expected accuracies, and Kappa. The mean confusion matrix of Table 5 shows the ground truth (columns) of the two approaches (rows). The main diagonal values evidence the imbalance between the number of ADLs and falls. This test provided an observed accuracy of 0.9945, and an expected accuracy of 0.3990 for a final Kappa value of 0.9908, which is significantly close to 1 (perfect measure).

A closer view on those specific activities that failed through the 10-fold validation showed 13 false negatives over six different activities, with four failures on F11 (fall backward when trying to sit down) and six failures on F13 (fall forward while sitting, caused by fainting or falling asleep). The test also showed 12 false positives, with six failures on D13 (sitting for a moment, lying quickly, waiting a moment, and siting again), and four on D14 (being on one’s back, changing to a lateral position, waiting a moment, and changing to one’s back again). The false positives were mainly on bed-related activities. This could be due to unnatural impacts with the pad, as it is harder than a mattress. The false negatives came mostly from the same subject, but their acceleration waveforms were not too different to be considered as outliers. As will be shown in the next section, these errors did not replicate in on-line validation.

### 4.3. On-Line Validation

In order to verify the off-line results presented in Table 4, we repeated the activities of SisFall with six young adults and an elderly person with the algorithm implemented on the device (see Section 2). During the tests, we verified on-line that the alarm was turned on (with an indicator incorporated to the device). Additionally, all raw data and the device computations were recorded in text files. We obtained no significant differences between the device and the computer. The proposed approach was implemented on the embedded device with the same parameters and sample frequency defined above (25 Hz). The threshold for $J_{3}$ was set at 40,000. The six volunteers performed 18 types of ADLs and 15 types of fall in the same way that the SisFall dataset indicated (around 100 total trials per subject).

The participants presented a total of 4 false positives and 1 false negative. Subject SE06 (the elderly person) did not show errors. All false positives were in D13 and D14 (bed-related ones). Following Figure 7, it is clear that these activities are commonly close to the threshold. A deeper analysis of this problem (which is not reflected in the following test) demonstrated that, when a person moves on the bed, it is common to separate the hip from the mattress and let it fall in the new position. The pad used for this experiment is harder than a mattress increasing the false positive probability. The overall results coincided with the statistics expected from Table 4.

### 4.4. Full-Day (Pilot) Tests

**Figure 8:**
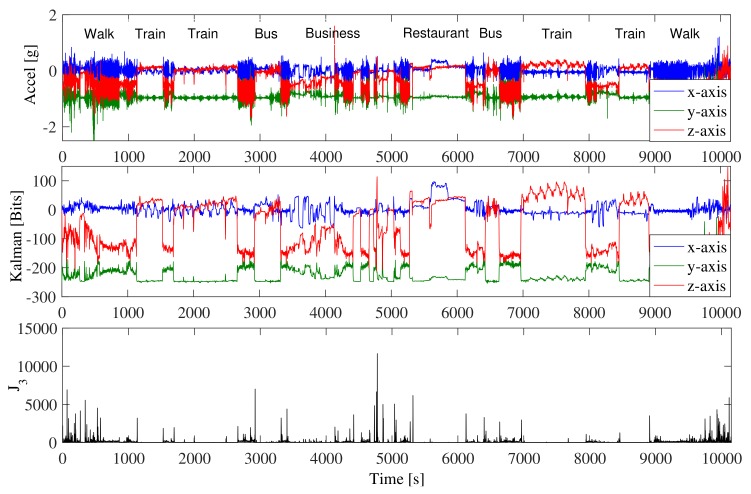
SM03’s trip downtown. (**top panel**) Raw acceleration data, 2 h and 45 min of recording. (**second panel**) First three states of the Kalman filter. (**third panel**) Feature $J_{3}$. It was always below the threshold (set at 40,000). Data recorded and processed with the embedded device of Figure 1.

We invited three independent elderly participants that were not part of SisFall acquisition (in order to avoid biases) to carry the device for full days (see Section 2). We asked them to behave normally while carrying the device during the day, and we checked the integrity of the devices every couple of hours. They used the device permanently except during night sleep and shower. The files were cut in segments to avoid computational overloads (one hour of recording implied a text file of approximately 10 MB). The following is a summary of the recorded activities:

- SM01 assisted in Tae-Bo dancing lessons for adults (INDER Medellín, Colombia) and stayed at home cooking, washing clothes, cleaning, and resting. She also made several trips downtown, walked on the street, and traveled by motorcycle.
- SM02 stayed at home most of the time and engaged in cooking, cleaning, and sitting in the dining room. She is a dressmaker, so she sits at home for long periods of time. She also traveled by bus sometimes, and during the last two days she was sick and rested at home.
- SM03 commuted to a business downtown and to a church. The rest of the time, he stayed at home in bed or in the dining room (reading). In Figure 8, we show one of his trips downtown (file SM03_1 of [34]). This trip included stairs, two train trips, and two bus trips. Note that, despite the wide amount of activities, the levels of feature $J_{3}$ were not close to the threshold (40,000).

We recorded approximately 170 h of uncontrolled ADLs divided in 77 text files. This dataset is available for download [34]. It includes raw acceleration data, low-pass-filtered data, the states of the Kalman filter, the three classification features ($J_{1}$, $J_{2}$, and $J_{3}$), and an indicator of falls detected. These data were recorded with three embedded devices. We additionally published a log of the activities performed by the participants and our explanation for every false positive.

**Figure 9:**
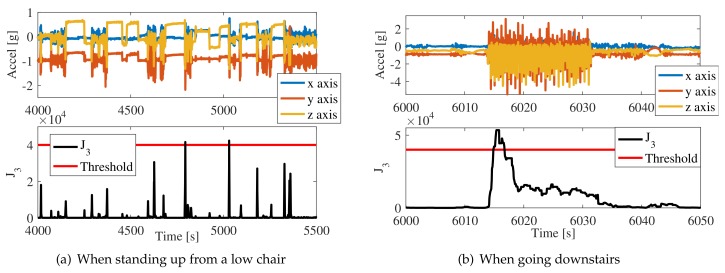
False positives. SM01 showed high accelerations for everyday activities. (**a**) Note that her accelerations when standing up from a low chair just crossed the threshold; (**b**) The periodicity detector did not work correctly when going downstairs because the subject suddenly started the activity and the Kalman filter requires a few steps to compute the period.

The behavior of our devices is presented as follows:

- SM01 had nine false positives during the recordings. Four of them were generated when she stood up from a low chair or from the sidewalk. As shown in Figure 9a, she would stand up fast and her acceleration was close to the threshold. Another two false positives were generated when going downstairs (see Figure 9b). The final three false positives were undetermined, but are presumed to be due to direct impacts to the device.
  The subject is an active person and overall, her movements showed accelerations close (and sometimes higher) to young adults. This behavior contradicts findings of [32]. Our findings suggest that independent elderly people may show the same accelerations in ADLs as do young adults. Consequently, simulating the ADLs of young people to obtain information about the uncontrolled ADLs of elderly people might be a better alternative to simulating ADLs with elderly people, who always show lower acceleration values.
- SM02 had a total of seven false positives. One was a false positive for sitting fast on a chair. Five other false positives were generated because she usually supported her belly against the kitchen or the table. She left her home several times, and twice the device was impacted and lost its SD card. This is worrying since, after an interview, we concluded that she strongly impacted the device in both cases, presumably against furniture. We presume that it was caused by her low height and by the shape of her belly (see Figure 1b). In order to solve this issue, we asked her to use the device on the inner side of the belt (i.e., with the *z*-axis pointing towards the back of the subject). After this modification, she did not show any more false positives.
- SM03 did not show false positives.

In total, there were 16 false positives in this validation test (divided in two subjects). It is interesting that the number of false positives decreases as age increases. Indeed, when we changed the direction of the device of SM02, she did not present more false alarms. If we consider all falls, this means approximately one false alarm every 10 h. This is a high frequency that can be reduced with some adjustments: (i) As shown in Figure 9a, we could slightly increase the threshold after some training for highly active elderly subjects; (ii) Once we put the device of SM02 on the inner side of the belt, she stopped hitting it against furniture. She also slightly adjusted the device to the right; we cannot determine the effect this had on the performance of the sensor during falls, but the data does not show significant differences in ADLs; (iii) None of the subjects presented false positives during trips (even when traveling by motorcycle). We consider this a measure of good performance. Finally, false alarms at the very beginning or end of the files were not considered for analysis, as they were caused by devices being turned on in non-vertical directions or being taken off the belt before the device is turned off.

## 5. Discussion and Conclusions

In this paper, we present a fall detection methodology with the following features: simple frequency filtering, a non-linear feature based on commonly used ones, threshold-based classification, and a periodicity detector to avoid false positives. With these features, we generated a novel fall detection algorithm centered on a Kalman filter stage and a non-linear classification feature. The Kalman filter is not computationally intensive, as it is Markovian, and it is shown here to be stable with acceleration data. We selected the Kalman filter because of its low computational cost and robustness; it provided an orientation level to a variance feature and at the same time a sinusoidal signal when the subject performed periodic activity. This last result highly reduced the computational cost to obtain the period of the signal, as it avoids more elaborate computations such as Wavelets or auto-correlation [19].

The most significant improvement of this approach consists in in how the combined non-linear feature ($J_{3}$) provided higher accuracy (99.4% with SisFall dataset) than the individual features (94.3% and 96.4%, respectively). We obtained this feature after analyzing individually several features with each activity (finally keeping the sum vector magnitude $J_{1}$ and standard deviation magnitude $J_{2}$). They were selected as they were highly complementary (each fails in different activities). The new non-linear feature used for this work was obtained in an intuitive way and, together with a threshold-based classifier, achieved a 99.4% accuracy with the SisFall dataset.

Our selection of the sum vector magnitude and standard deviation magnitude as base functions for our non-linear feature was not arbitrary. Many sets of features will achieve similar results, but these two features alone can accomplish our requisites. However, implementing this approach on different devices (such as smartphones) may require a different set of basis features. Moreover, three main considerations need to be made: the smartphone is usually positioned in a pocket near the waist, and this lateral repositioning could affect the fall detector, so the selected features should account for it. Second, although the periodicity detector can work when the device is upside down (it removes the bias of the *y*-axis), it will not work correctly if the device is lateral (the *x*-axis is pointing up or down); however, this condition is not common, and the Kalman filter could be adapted to follow the gravity direction instead of the *y*-axis direction. Finally, we need to recognize whether the device is active (on the user’s hand) to avoid false positives due to handling.

We implemented this methodology in embedded devices and tested it by redoing on-line all SisFall activities. We then validated our work with full-day tests with the target population (two female and one male, all over 60 years old). We asked them to do what they usually do, including traveling by train and bus, doing exercise, and cooking or cleaning. The devices behaved as expected, with some false positives when subjects went downstairs, stood up from a low chair, and directly struck the device. This final cause of false positives is out of the scope of this work and a good starting point for future analysis.

This final validation demonstrated that the proposed methodology can be used with the target population. We recorded and released [34] more than 170 h of ADL recordings with the target population under uncontrolled conditions. This is to our knowledge the largest validation dataset used for a fall detection approach. However, only real falls that may occur at any moment will show the real accuracy of our approach.

This extensive validation additionally allowed us to observe how the devices behaved in terms of power consumption. On average, one battery charge lasted between four and five days (with the device turned off when the subjects were sleeping at night) under commercial use configuration (i.e., without recording data). We point out that this result is not conclusive or subject to comparison because it mostly depends on the battery characteristics and hardware configuration, and these data are not usually released by authors. However, this result demonstrates that our device accomplishes with the full-day single-charge requirement for being feasible under real-life use. Moreover, we consider that our approach is energy-efficient for the following reasons: (i) Sampling at 25 Hz instead of the usual 50–100 Hz implies being active for less time; (ii) With respect to the number of sensors, the gyroscope, for example, consumes several times the current of the accelerometer (hundreds of μA compared to tens of μA), i.e., having only one triaxial accelerometer avoids extra consumption; Finally, (iii) the Kalman filter is not computationally intensive, and using a threshold-based classification is optimal in terms of computing load. Compared with neural networks or support vector machines, our approach is significantly more efficient [9]. 

## Figures and Tables

**Table 1:** Gender, age, height, and weight of the on-line test participants.

Code	Gender	Age	Height [m]	Weight [kg]
SM01	Female	61	1.56	54
SM02	Female	70	1.46	56
SM03	Male	81	1.62	68

**Table 2:** Current consumption of commercial embedded gyroscope and accelerometer sensors under normal operating conditions.

Brand	Device	Accelerometer	Gyroscope	Max Total
Analog Devices	ADXC1501	N/A	N/A	<16 mA
InvenSense	MPU-9250	8 μA	3.2 mA	6 mA
ST	LSM6DS3	70 μA	N/A	900 μA
BOSCH	BMI160	180 μA	850 μA	990 μA

**Table 3:** Test on the SisFall dataset without a periodicity detector.

	$J_{1}$	$J_{2}$	$J_{3}$
Sensitivity [%]	92.92 ± 1.56	96.06 ± 1.52	99.27 ± 0.78
Specificity [%]	81.72 ± 2.22	96.79 ± 1.12	99.37 ± 0.36
Accuracy [%]	86.14 ± 1.36	96.50 ± 0.84	99.33 ± 0.28
Threshold	110.88 ± 3.23	22.88 ± 0.027	42,628 ± 511.59

**Table 4:** Test on the SisFall dataset with the periodicity detector.

	$J_{1}$	$J_{2}$	$J_{3}$
Sensitivity [%]	97.35 ± 1.37	96.15 ± 1.59	99.28 ± 0.59
Specificity [%]	91.49 ± 1.74	96.69 ± 1.30	99.51 ± 0.48
Accuracy [%]	94.42 ± 1.33	96.42 ± 0.58	99.39 ± 0.36
Threshold	103.03 ± 0.02	22.914 ± 0.11	42,230 ± 985.01

**Table 5:** Confusion matrix for 10-folds of validation (451 files each).

		Ground Truth	
		ADL	FALL
Proposed	ADL	269.8±9.56	1.3±1.25
Approach	FALL	1.2±0.92	178.7±9.68
